# Heavy Metal-Contaminated Soils and Gastric Cancer Risk: Molecular Insights and the Relevance of a One Health Perspective

**DOI:** 10.3390/ijms262311526

**Published:** 2025-11-27

**Authors:** Claudia Reytor-González, Sonia Emilia Leyva Ricardo, Yasniel Sánchez Suárez, Vianey Ariadna Burboa Charis, Emilia Jiménez-Flores, Emilia Cevallos-Fernández, Martín Campuzano-Donoso, Daniel Simancas-Racines

**Affiliations:** 1 Escuela de Medicina, Pontificia Universidad Católica del Ecuador, Santo Domingo 230203, Ecuador; 2 Facultad de Ciencias de la Ingeniería e Industrias, Universidad UTE, Santo Domingo 230208, Ecuador; sonia.leyva@ute.edu.ec; 3 Centro de Estudios Futuro, Proyecto de Desarrollo Local RUTA FUTURO, Universidad de Matanzas, Matanzas 40100, Cuba; yasnielsanchez9707@gmail.com; 4 Departamento de Biotecnología y Ciencias Alimentarias, Instituto Tecnológico de Sonora, Obregon 85000, Sonora, Mexico; vburboach@gmail.com; 5 Centro de Investigación en Salud Pública y Epidemiología Clínica (CISPEC), Facultad de Ciencias de la Salud Eugenio Espejo, Universidad UTE, Quito 170129, Ecuador; mariae.jimenez@ute.edu.ec; 6 Centro de Investigación de Alimentos (CIAL), Facultad de Ciencias de la Ingeniería e Industrias, Universidad UTE, Quito 170129, Ecuador; cfel46261@ute.edu.ec; 7 Independent Researcher, Quito 170102, Ecuador; martincd01@hotmail.com; 8 Facultad de Salud y Bienestar, Pontificia Universidad Católica del Ecuador, Quito 170143, Ecuador

**Keywords:** heavy metals, gastric cancer, cadmium, arsenic, lead, environmental contamination, One Health

## Abstract

Heavy metal contamination in agricultural soils has emerged as a critical environmental and public health issue associated with increased gastric cancer incidence worldwide. Among the most concerning pollutants are cadmium, arsenic, and lead, which persist in the environment and enter the human body primarily through the soil–plant–food chain. This review integrates environmental, molecular, and epidemiological evidence to explain how these metals alter gastric mucosal biology and promote carcinogenesis. Mechanistically, cadmium, arsenic, and lead trigger oxidative stress, mitochondrial dysfunction, DNA damage, and epigenetic reprogramming, resulting in genomic instability, resistance to programmed cell death, and the transformation of epithelial cells into invasive phenotypes. These molecular disruptions interact with *Helicobacter pylori* infection, microbial imbalance, chronic inflammation, and hypoxia-driven remodeling of the gastric stroma, all of which enhance angiogenesis and tumor progression. Advanced experimental platforms, such as gastric organoids, immune co-cultures, and humanized animal models, are improving the understanding of these complex interactions. Adopting a One Health perspective reveals the continuity between environmental contamination, agricultural production, and human disease, underscoring the importance of integrative monitoring systems that combine soil and crop analysis with molecular biomarkers in exposed populations. Strengthening this interdisciplinary approach is essential to design preventive strategies, guide remediation policies, and protect human, animals, and environmental health.

## 1. Introduction

Gastric cancer remains a major global health challenge, ranking among the top causes of cancer mortality and showing clear geographic clustering that points to environmental drivers of risk [1,2,3,4]. Emerging evidence places contaminated soils, particularly with cadmium (Cd), arsenic (As), and lead (Pb), at the center of a soil–plant–human exposure continuum that can modulate gastric carcinogenesis [5,6]. This review foregrounds molecular insights into how those metals perturb gastric mucosal biology and argues for a One Health lens that integrates environmental, animal, and human health data to guide prevention [4,7,8]. Recent syntheses and epidemiologic studies underscore the problem’s scope: soil metal burdens correlate with stomach cancer patterns in industrial, mining, and peri-urban settings, while dietary pathways (notably rice and vegetables grown on polluted soils) often dominate exposure in the general population [2,3,5,6,9].

A molecular focus is warranted because heavy metals perturb multiple cancer hallmarks in gastric epithelia [7,10]. Mechanistically, Cd, As, and Pb amplify oxidative stress [11,12,13], derail deoxyribonucleic acid (DNA) repair [7,13], shift epigenetic programs [14,15], and interfere with cell death pathways [16,17], thereby fostering genomic instability and malignant progression [7,12]. These effects are supported by in vitro models [18,19], animal work [20], and molecular epidemiology linking metal burdens to tumor features (e.g., microsatellite instability, receptor signaling) in gastric tissue [8,21].

At the same time, a narrow toxin-host paradigm is insufficient, because the gastric niche is both immunologically and microbially active, and metal exposures interact with co-factors that shape disease trajectories [22,23,24]. For example, *Helicobacter pylori* (*H. pylori*) initiates chronic gastritis and atrophy; as pH rises and glandular architecture changes, the gastric microbiota becomes dysbiotic, often enriched for oral/intestinal taxa with nitrosating functions, which can potentiate inflammation and genotoxic stress [25,26,27]. Incorporating this microbial dimension clarifies why only a subset of exposed individuals develops cancer and highlights feedback loops between environment, host, and microbes [22,28].

A One Health framework provides the systems-level scaffolding needed to translate these insights. It connects soil chemistry (speciation, bioaccessibility), plant uptake (root transporters, rhizosphere conditions), food webs, livestock, and human diets, while embedding surveillance, risk modeling, and policy [4,29,30]. It operationalizes speciation and bioaccessibility testing for priority metals in soils [31], links them to crop uptake mechanisms and agronomic controls [5], and couples source-apportioned risk and stochastic exposure models to target interventions [32,33,34]. In agricultural systems, soil pH, organic matter, and metal speciation drive bioavailability and transfer; site-specific assessments in mining and peri-urban districts illustrate how these factors shape human risk [32,35]. Crops such as rice can accumulate As and Cd to intake-relevant levels, especially where irrigation water is contaminated, making the soil–plant pathway a dominant exposure route in many regions [5,6]. These realities argue for integrative risk assessments that pair environmental monitoring (soil, water, crops) with biomonitoring and mechanistic biomarkers in exposed communities, including blood/tissue metal burdens and tumor-linked molecular features [8,21,36,37].

The objective of this review is to critically synthesize the molecular mechanisms by which Cd, As, and Pb from contaminated soils drive gastric carcinogenesis; to delineate how co-factors within the gastric microenvironment—including *H. pylori* infection, dysbiotic microbiota, innate immune signaling, hypoxia-driven angiogenesis, and stromal remodeling—amplify or modulate metal toxicity; and to argue that prevention and policy must adopt an integrated One Health approach that spans soil, crops, livestock, and human populations. To this end, we integrate environmental data (soil and crop contamination profiles, metal speciation and bioaccessibility, soil-to-plant transfer, and multi-pathway exposure modeling), molecular evidence (oxidative stress, DNA damage and repair dysfunction, epigenetic alterations, and oncogenic signaling), and epidemiology, while highlighting critical research gaps, such as metal mixtures, cumulative exposures, and limited longitudinal data, and the translational levers needed for surveillance, risk assessment, and targeted remediation.

## 2. Methodology

For this narrative review, we conducted a comprehensive search in PubMed, Scopus, Cochrane Library, and Epistemonikos for publications from 2005 to 2025. Search terms combined concepts related to “cadmium,” “arsenic,” “lead,” “soil contamination,” “gastric carcinogenesis,” and “One Health”.

Two reviewers (CR-G and S.E.L.R.) screened titles and abstracts independently, selecting studies for full review only when both agreed on their relevance. The reference lists of included articles were also examined to identify additional relevant publications. We incorporated mechanistic studies, environmental assessments, epidemiological evidence, and reviews that contributed to understanding the soil–plant–human continuum and its molecular implications for gastric cancer.

## 3. Sources and Environmental Persistence of Heavy Metals in Soil

Soil contamination with heavy metals is a critical threat to ecosystems [38,39] and global food security [40,41,42]. Major anthropogenic sources include mining, which generates tailings and waste rich in trace elements [43,44]; industry, which releases atmospheric particles and effluents [45]; and intensive agriculture, where fertilizers, sewage sludge, and pesticide residues have contributed to long-term soil accumulation [46,47].

Among these contaminants, Cd, As, and Pb are of greatest concern due to their toxicity and persistence [48]. Cd is easily absorbed by plant roots [49]; as occurs in forms with variable toxicity [50]; and Pb binds strongly to soils but can be mobilized under acidic conditions [50]. This makes food crops the main entry point into the human food chain [51,52].

### 3.1. Mining, Industry, and Agriculture

Mining, particularly for base metals and coal, is the most significant source of heavy metal contamination. Waste rock and tailings produce acid mine drainage, which mobilizes Cd, Pb, As, and other metals into nearby soils and waters [53,54]. Industrial emissions from smelters, cement plants, and incinerators spread metals through atmospheric deposition over large areas [55].

Agriculture represents a chronic diffuse source. Phosphate fertilizers are a major pathway for Cd [53]. Biosolids enrich soils with nutrients but also introduce metals when poorly treated. Historic pesticides and fungicides left residues of As, Pb, and Cu, especially in vineyards and orchards [56]. Irrigation with contaminated water further contributes to accumulation (Table 1) [57].

### 3.2. Environmental Persistence and Ph-Driven Bioavailability

The persistence and mobility of Cd, As, and Pb in soils are strongly governed by soil pH, redox conditions, and sorption onto mineral and organic components. Cd is consistently identified as the metal whose bioavailability is most sensitive to acidic pH [60]. Evidence shows that Cd availability increases in soils with pH < 6, where reduced sorption to carbonates, oxides, and organic matter enhances its mobility [61]. n calcareous and agricultural soils, higher pH conditions (generally pH > 7) promote Cd immobilization, lowering the fraction available for plant uptake.

As displays a distinct behavior, as its speciation depends on both pH and redox potential. Under oxidizing and moderately acidic to neutral conditions (pH 5–7), arsenate [As(V)] predominates and is strongly adsorbed to Fe/Al oxides, resulting in lower bioavailability. In contrast, alkaline (pH > 7) or reducing environments favor the formation of arsenite [As(III)], which is more mobile and more readily taken up by plants, particularly in flooded or anaerobic soils used for rice cultivation [62].

Pb remains the least mobile and least bioavailable of the three metals under most agricultural conditions. Pb forms stable complexes with clays, Fe/Mn oxides, and organic matter, leading to limited movement across typical soil pH ranges (~5.5–7.5) [63]. Although several studies show that Pb solubility increases under strongly acidic conditions (pH < 5), its mobilization remains substantially lower than that of Cd or As [60].

**Figure 1:**
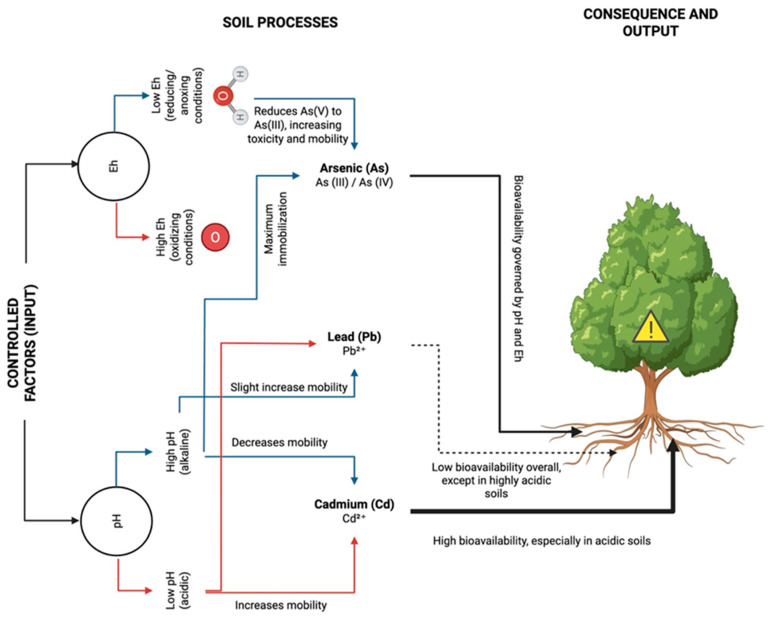
Influence of Soil pH and Redox Potential on Cadmium, Arsenic, and Lead Speciation, Mobility, and Plant Bioavailability. Acidic soils (low pH) increase Cd mobility and bioavailability, whereas alkaline conditions (high pH) enhance As mobility through arsenate [As(V)] reduction to arsenite [As(III)] under reducing conditions. Lead (Pb^2+^) remains largely immobile, though its mobility slightly increases in acidic or oxidizing environments. Overall, the bioavailability of these metals to plants is strongly governed by pH and Eh, with acidic soils posing the greatest risk for Cd uptake and anoxic conditions increasing the toxicity of As.

Collectively, current evidence shows that Cd bioavailability increases markedly in acidic soils, As mobility rises under alkaline or reducing conditions, and Pb remains strongly sorbed except under extreme acidity. These distinctions are central to understanding soil–plant transfer and exposure potential across contaminated agricultural landscapes. Figure 1 summarizes how these factors influence metal mobility and plant bioavailability.

### 3.3. Implications for Human Health

The long half-life of Cd, As, and Pb in soils means both past and present contamination will impact ecosystems and health for generations. Human exposure occurs mainly through consumption of contaminated crops [64,65,66,67], drinking water percolated through polluted soils [68], and inhalation of resuspended dust, a critical route for Pb exposure near industries and roads [69,70].

Addressing this challenge requires integrated strategies: ongoing monitoring and risk assessment, safe agricultural practices (e.g., pH control, phytoremediation, low-risk crops), remediation of hotspots, and stronger emission controls. Future research should focus on molecular-level mechanisms of bioavailability and cost-effective soil management to prevent contaminated soils from reaching the food chain.

## 4. Molecular Mechanisms of Heavy Metal-Induced Gastric Carcinogenesis

**Figure 2:**
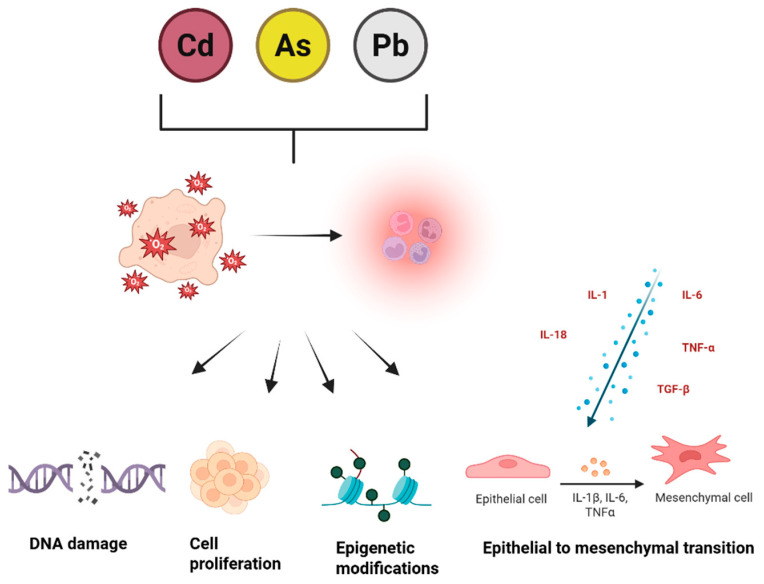
Molecular mechanisms underlying heavy metal-induced tumorigenesis. A crucial factor in their carcinogenic potential is the ability to drive persistent inflammation, which acts as a bridge between exposure and tumor formation. Chronic inflammation enhances DNA damage, fuels cell proliferation, weakens repair systems, and promotes epithelial–mesenchymal transition (EMT), enabling greater invasiveness and metastatic potential. This state is further reinforced by cytokines such as IL−1, IL−6, IL−18, TNF-α, and TGF-β, with IL−1 shifting from its usual protective function to one that facilitates cancer progression under sustained inflammatory conditions [72].

Heavy metals represent a major class of environmental pollutants that contribute to chronic disease risk. Among them, Cd, As, and Pb are particularly harmful, as they trigger oxidative stress, impair mitochondrial activity, and induce genetic as well as epigenetic changes [71]. In addition to these toxic effects, these metals contribute to cancer development through multiple interconnected mechanisms (Figure 2).

### 4.1. Cadmium

Cd is a widespread environmental contaminant and a category I human carcinogen that accumulates in tissues due to its prolonged biological half-life and limited excretion [73]. Major sources of human exposure include contaminated food, soil, and tobacco smoke. Although Cd is not directly redox-active, it indirectly elevates reactive oxygen species (ROS) levels by impairing mitochondrial function and reducing the activity of key antioxidant defenses, including glutathione (GSH), superoxide dismutase (SOD), and glutathione peroxidase (GPx) [74]. Cd disrupts electron transport chain complexes I and II, leading to increased mitochondrial ROS production, lipid peroxidation, protein oxidation, and DNA damage, while simultaneously decreasing ATP synthesis and promoting a shift toward anaerobic metabolism [75]. Additionally, Cd binds to thiol groups and displaces essential metals, further inhibiting antioxidant enzymes, exacerbating oxidative stress, and disturbing cellular redox homeostasis, ultimately impacting all major biomolecules [76].

Cd compromises genomic stability by interfering with DNA repair pathways, including inhibition of poly(ADP-ribose) polymerase (PARP), nucleotide excision repair enzymes, and mismatch repair complexes, resulting in accumulation of mutations [77]. It destabilizes the E3 ubiquitin ligase RNF168, impairing histone ubiquitination and preventing the recruitment of essential repair factors such as 53BP1 and BRCA1, which indirectly disrupts ATM-mediated signaling and partially abrogates the G2/M checkpoint, allowing cells to enter mitosis with unrepaired DNA [77,78]. Moreover, Cd downregulates the tumor suppressor p53, reducing DNA damage-induced cell cycle arrest and apoptosis, thereby permitting survival and proliferation of genetically altered cells [76].

Beyond its effects on DNA, Cd also exerts epigenetic influences that contribute to carcinogenesis. It promotes hypermethylation and transcriptional silencing of tumor suppressor genes, including p16^INK4a, RASSF1A, and E-cadherin, alters histone acetylation and methylation patterns, and dysregulates microRNAs by enhancing oncogenic miR-21 expression while suppressing tumor-suppressive miR-34a, collectively fostering abnormal cell proliferation and resistance to apoptosis [79]. These epigenetic changes, combined with oxidative stress and impaired DNA repair, facilitate epithelial–mesenchymal transition (EMT), increasing cancer cell invasiveness, motility, and aggressiveness [80]. Cd further modulates stress-response genes, metallothioneins, heat shock proteins, and transcription factors such as NF-κB, NRF2, and MTF1, disrupting cellular regulatory networks and promoting malignant transformation [81,82].

### 4.2. Arsenic

As, a naturally occurring metalloid found in soil, water, and food, represents a significant health hazard, particularly through exposure to contaminated groundwater. Its association with cancer, diabetes, hypertension, neurodegenerative conditions, and skin disorders imposes substantial health and economic challenges, underscoring the importance of investigating its mechanisms of carcinogenicity [83].

As exerts its carcinogenic and cytotoxic effects through a multifaceted network involving modulation of signaling pathways, oxidative stress, epigenetic alterations, and apoptosis regulation. A primary target of As is the PI3K/AKT/mTOR signaling cascade, which controls cell growth, proliferation, metabolism, and survival. As exposure stimulates upstream receptors, such as EGFR, thereby activating PI3K and AKT, which in turn promotes mTOR signaling and the activity of transcription factors including HIF-1, AP-1, FOXO, and NF-κB [84]. These events collectively facilitate cellular proliferation, anchorage-independent growth, and malignant transformation. Concurrently, As influences MAPK pathways, including ERK1/2, JNK, and p38, in a time- and dose-dependent fashion, with biphasic or temporally distinct activation patterns that contribute to either apoptotic or proliferative cellular responses depending on cell type and exposure duration [85,86].

Oxidative stress is a key mediator of As-induced toxicity, with mitochondria serving as a principal source of ROS. Elevated ROS levels impair mitochondrial function by disrupting respiratory complexes, causing cristae damage and swelling, and activating PINK1/Parkin-dependent mitophagy. While mitophagy can protect cells by removing damaged mitochondria, excessive ROS promotes apoptosis. ROS also oxidize cysteine residues in proteins, impairing DNA repair enzymes and modifying transcription factors such as AP-1, NF-κB, and Nrf2. As activates Nrf2 via both canonical and noncanonical routes, including inhibition of Keap1, accumulation of p62, and Nrf2 acetylation, creating a positive feedback loop. Although Nrf2 activation initially mitigates oxidative stress, chronic stimulation can enhance cell survival, metabolic adaptation, and apoptosis resistance, promoting oncogenic transformation [86].

As directly induces apoptosis by activating intrinsic and extrinsic caspase pathways. Caspase-8 and -9 are crucial for initiating cell death, while caspase-3 cleavage shows a complex regulatory pattern [87]. Morphological changes, G2/M cell cycle arrest, and cytoskeletal disruption accompany these effects, with sodium arsenite demonstrating substantially higher potency than dimethylarsenic acid [88]. As also downregulates pro-apoptotic genes, including BAX and caspases, while shifting the Bax/Bcl-2 ratio toward apoptosis, contributing to cytotoxicity in diverse cell types [89]. Fas signaling also mediates apoptotic responses, highlighting the coordinated regulation of multiple pathways [85,90].

Epigenetic dysregulation further contributes to As-induced carcinogenesis. As alters histone modifications, decreasing H4K16Ac, H2BK120ub, and H3K9me3 while increasing H3K4me2/3, H3K9me2, H3K27me3, H3S10 phosphorylation, and γH2AX, affecting chromatin accessibility and proto-oncogene activation [91]. Simultaneously, DNA methylation is disrupted through SAM depletion, DNMT downregulation, Tet inhibition, and altered CTCF binding, resulting in global hypomethylation and promoter-specific hypermethylation of tumor suppressor and DNA repair genes such as MLH1, ERCC1, and ERCC2. By integrating MAPK and PI3K/AKT activation, ROS generation, mitochondrial dysfunction, suppression of apoptotic genes, and epigenetic modifications, As promotes genomic instability and drives malignant transformation.

### 4.3. Lead

Pb is a toxic, odorless, silver-bluish-white heavy metal that accumulates in body tissues over time, causing serious systemic health effects [92]. Recognized as “probably carcinogenic to humans” (group 2A) by the IARC, Pb exerts both direct and indirect cellular effects that contribute to disease [93]. A primary mechanism of toxicity involves disruption of intracellular calcium (Ca^2+^) homeostasis. Calcium is a critical signaling ion regulating processes such as cell proliferation, apoptosis, migration, and immune function, all of which are fundamental for cancer initiation and progression [94,95]. Pb interferes with the tightly regulated distribution of calcium among the endoplasmic reticulum (ER), mitochondria, and lysosomes, and inhibits enzymes involved in vitamin D activation, such as 1α-hydroxylase, thereby lowering serum calcium levels [92,96]. These disturbances impair ER-centered calcium signaling and mitochondrial function, promoting abnormal cell growth and resistance to programmed cell death [97].

In addition to calcium dysregulation, Pb contributes to genotoxic stress. Human studies indicate that occupational exposure is linked to chromosomal aberrations, DNA strand breaks, and micronucleus formation [98]. Indirect mechanisms include ROS overproduction, inhibition of DNA repair pathways such as base excision repair (BER) and nucleotide excision repair (NER), replacement of zinc in DNA-binding proteins, and covalent interactions that alter DNA structure [13]. These combined effects compromise genome integrity and may dysregulate tumor suppressor and promoter genes, supporting carcinogenic processes [13,93].

Chronic inflammation is another central feature of Pb toxicity. Pb modulates innate and adaptive immunity, alters cytokine profiles, and increases ROS generation, causing damage to lipids, proteins, and DNA [99]. Exposure elevates cytokines including IL-1β, IL-12p70, and IFN-γ (HUO), while impairing T and B lymphocyte and NK cell function, which are essential for anti-cancer defense. These immune and oxidative effects create a microenvironment favorable to malignant transformation [4,100].

Pb also disrupts gut microbiota, representing an additional pathway for chronic inflammation and disease. Exposure reduces bacterial diversity and shifts the composition of key phyla such as Firmicutes, Bacteroidetes, and Proteobacteria. At finer taxonomic levels, potentially pro-inflammatory families including Erysipelotrichaceae, Desulfovibrionaceae, and Porphyromonadaceae are enriched, whereas beneficial genera such as *Akkermansia*, *Bacteroides*, and certain *Lachnospiraceae* are diminished [101]. These microbial changes compromise intestinal barrier function and intensify inflammation. Metabolically, Pb reduces levels of short-chain fatty acids (SCFAs) like acetate, propionate, and butyrate, disrupts bile acid metabolism, depletes antioxidants such as vitamin E, and alters amino acid and nitrogen pathways, further sustaining oxidative stress and chronic low-grade inflammation [102]. Such mechanisms may indirectly link Pb exposure to gastric inflammation and cancer risk.

## 5. Gastric Microenvironment and Co-Factors

### 5.1. Microbial–Immune Crosstalk That Shapes Metal Toxicity

The gastric mucosa is an immunologically and microbially dynamic niche in which metals do not act in isolation. *H. pylori* establishes chronic inflammation, atrophy, and intestinal metaplasia, and as acid output declines the microbial community shifts toward oral and intestinal taxa with potential nitrosating capacity—a configuration repeatedly observed in sequencing studies of gastric cancer—with increased ROS/reactive nitrogen species (RNS) burden [103,104,105]. This dysbiotic state, characterized by reduced diversity and loss of *Helicobacter* dominance, favors nitrate–nitrite metabolism, generates reactive nitrogen species, and potentiates oxidative and genotoxic stress in the epithelium tight junctions [106,107]. Against this backdrop, Cd, As, and Pb can accelerate barrier dysfunction, blunt antioxidant defenses, and tilt redox homeostasis, thereby amplifying mutational pressure and epigenetic drift within metaplastic glands [108,109,110].

Innate immune sensors provide a mechanistic bridge between microbial cues, tissue stress, and metal exposure. Pattern-recognition receptors (PRRs), notably Toll-like receptors (TLRs), integrate microbial products and damage-associated signals to activate nuclear factor kappa B (NF-κB) and, via interleukin-6/Janus kinase signaling pathway (IL-6/JAK) signaling, signal transducer and activator of transcription 3 (STAT3), sustaining cytokine fields (tumor necrosis factor alpha (TNF-α), IL-6, IL-8) that promote epithelial proliferation, survival, and invasion [111,112,113,114,115]. In vitro, conditioned media from LPS-activated macrophages accelerates gastric cancer cell growth and triggers concurrent NF-κB/STAT3 activation via inhibitor of kappa B alpha (IκBα) degradation and JAK2 phosphorylation, while also reshaping the tumor epigenome [116,117]. Metals intensify these circuits by increasing mitochondrial ROS, inhibiting DNA repair enzymes, and skewing apoptotic checkpoints, thereby lowering the threshold for PRR-driven transcriptional programs to translate into malignant advantage [109,110].

Inflammasome signaling adds a further layer of innate control that metals can perturb. The NOD-like receptor protein 3 (NLRP3) inflammasome coordinates caspase-1–dependent maturation of IL-1β and IL-18, reshaping epithelial integrity and immune infiltration in gastrointestinal tissues; metal-induced mitochondrial stress, lysosomal injury, and ROS are canonical NLRP3 triggers, and in the metal-exposed, *H. pylori*-colonized stomach these signals likely converge [118,119,120,121,122]. By coupling dysbiotic metabolites, bacterial ligands, and metal-derived danger cues, the inflammasome can push the microenvironment toward chronic, proliferative inflammation [103,119]. Taken together, these microbial–immune interfaces represent synergy points through which Cd, As, and Pb translate persistent inflammation into clonal selection and field cancerization [123,124,125,126].

### 5.2. Hypoxia, Angiogenesis, and Stromal Remodeling as Amplifiers of Metal Effects

Hypoxia is a defining spatial feature of gastric tumors that reprograms the microenvironment toward progression and therapy resistance. The transcription factor hypoxia-inducible factor-1α (HIF-1α) orchestrates angiogenesis, metabolic rewiring, extracellular matrix (ECM) remodeling, and immune evasion, and high HIF-1α expression in gastric cancer correlates with greater microvessel density, nodal metastasis, and advanced stage [127,128,129].

Metals intersect with this axis at multiple nodes. By increasing ROS, Cd and As can inhibit prolyl-4-hydroxylases that normally hydroxylate HIF-1α and target it for degradation, thereby stabilizing HIF-1α even when oxygen is present [130,131,132]; downstream, vascular endothelial growth factor (VEGF) and other pro-angiogenic mediators rise [127,133], while glycolytic, acid-tolerant phenotypes become favored in patchy oxygen landscapes [134,135]. These adaptations not only expand aberrant vasculature but may also facilitate delivery and retention of circulating metals and nitrosating precursors within tumor tissue, closing an exposure–biology loop [127,129,130].

Cancer-associated fibroblasts (CAFs) translate hypoxic signals into structural and immunologic change. As hypoxia sensors, CAFs remodel ECM architecture, increase matrix metalloproteinases (MMPs), and deposit aligned collagen tracks that lower the physical barrier to invasion; HIF-1α–responsive programs in CAFs also foster angiogenesis and metabolic symbiosis, including lactate shuttling that supports cancer cell growth under oxidative stress [129,136]. In metal-exposed settings, ROS–HIF coupling can exaggerate these functions: Cd and Ar can heighten oxidative tone, tilt transforming growth factor beta (TGF-β) signaling, and amplify MMP expression, while Pb’s interference with calcium homeostasis can modulate contractility and stromal–epithelial signaling [131,135,137,138]. Crosstalk with macrophages further reinforces this state: CAF-derived chemokines attract and polarize myeloid cells that, in turn, sustain STAT3/NF-κB-driven inflammation and release additional MMPs, a pattern reproduced in gastric cancer cell–macrophage systems [136,139]. Collectively, hypoxia, aberrant angiogenesis, and stromal plasticity provide a permissive biome through which metals translate environmental exposure into invasive phenotypes.

### 5.3. Platforms and Exposure Context to Interrogate and Mitigate Microenvironment–Metal Synergy

Model systems are increasingly capable of capturing metal–microenvironment interactions with high fidelity. Gastric organoids recapitulate epithelial lineage diversity and polarity and permit controlled exposure to Cd, As, or Pb while quantifying redox injury, DNA repair competence, and fate decisions. Patient-derived organoids (PDOs) maintain histologic and genomic features of source tumors and enable drug screening and biobanking [140]. Induced pluripotent stem cell (iPSC)-derived gastric organoids are useful to model early disease initiation and *H. pylori*-driven transformation [141,142]. Emerging 3D bioprinting with gelatin methacryloyl (GelMA) hydrogels yields gastric cancer constructs that reproduce proliferation, invasion, angiogenesis, and Warburg-like metabolism, with promise for high-throughput testing [143].

Co-culture approaches add stromal and immune context. Autologous gastric patient-derived organoids (PDO)/immune co-cultures predicting targeted-therapy efficacy and probing myeloid-derived suppressor cells (MDSC) function have been demonstrated [144]. Overlay methods that let organoids retain 3D structure while immune cells migrate freely enable real-time visualization of immune attack [145]. Peripheral blood mononuclear cell (PBMC)–organoid platforms extend this to evaluate immunotherapies, including chimeric antigen receptor (CAR)-engineered lymphocytes [146]. Recent organoid–immune co-culture work in metastatic disease underscores feasibility for prospective precision applications [147]. In vivo, mouse models bridge mechanisms and disease behavior. Orthotopic xenografts created by direct implantation of tumor cell suspensions achieve high engraftment and metastatic rates with shorter timelines versus fragment implantation [148]. Patient-derived orthotopic models recapitulate molecular/phenotypic features of human gastric adenocarcinoma and are especially informative for peritoneal carcinomatosis [149]. Humanized models reveal how uninvolved peritoneum becomes tumor-permissive during transcoelomic spread, highlighting emergency medical technician, stromal/immune infiltration, and inflammatory responses [150].

An exposure context overlays these platforms. Heavy metals relevant to gastric cancer, Cd, Ar, and Pb, drive ROS generation, weaken antioxidant defenses, disrupt repair and apoptosis, and activate inflammatory cascades [12,137]. Cd in particular has been linked to oral and gastrointestinal carcinomas via oxidative stress and DNA damage mechanisms [151]. Combined low-level metal exposures associate with systemic inflammation. Translationally, integrating exposure with mechanistic readouts (e.g., HIF-1α/VEGF, NF-κB/STAT3 targets, inflammasome activation, ECM/MMP programs) and with organoid/co-culture/mouse workflows [105,152,153] can pinpoint intervention nodes and accelerate prevention-to-therapy pipelines.

A One Health lens situates these mechanisms within real exposure chains from soil to stomach. In agricultural settings, master variables such as soil pH and organic matter govern metal speciation and the size of bioavailable pools, with pH, ionic strength, and dissolved organic carbon jointly determining solubility, activity, and complexation; speciation in turn controls plant toxicokinetics while toxicodynamics drives tissue injury [154,155]. Root uptake kinetics and rhizosphere chemistry—including pH, oxygenation, exudates, and microbiome activity—modulate transfer to edible tissues [156]. Irrigation with contaminated groundwater magnifies accumulation, especially in flooded rice systems where anaerobiosis favors As mobilization; rice can materially contribute to daily As and Cd intake, with reports of grain As exceeding health-protective limits and Cd bioaccessibility averaging approximately 24 percent [157,158,159,160]. Vegetables grown on peri-urban and mining-adjacent soils add additional dietary burdens, with transfer factors near unity and frequent exceedances of WHO/FAO or national standards [161,162]; children are particularly vulnerable where direct soil contact is appreciable [162]. Beyond diet, inhalation of contaminated dusts and dermal contact contribute to aggregate dose in occupational and some residential contexts, although pathway importance typically follows: contaminated produce > incidental soil ingestion > dermal contact > particle inhalation [162,163]. Source-apportioned risk models (e.g., Positive Matrix Factorization (PMF), Absolute Principal Component Score/Multiple Linear Regression (APCS-MLR)) and probabilistic Monte Carlo assessments link soil and crop measurements to intake distributions, attribute contributions to industrial activity, traffic, natural background, and mining (illustratively approximately 53, 18, 17, and 10 percent, respectively), and identify populations most likely to exceed health-protective thresholds [37,164,165,166]. These exposure insights map directly onto microenvironmental biology—oxidative stress, DNA-repair derailment, nitrosation chemistry, hypoxia stabilization, and stromal activation—while pointing to practical levers: water-management regimes that limit Ar uptake and cultivar selection to lower Cd accumulation [160,167]. Key microenvironmental co-factors, their metal-linked effects, pathways, and translational readouts are summarized in Table 2.

## 6. One Health Perspective: Connecting Environmental and Human Health

The One Health approach highlights the intricate interdependence of human, animal, and environmental health, underscoring the need for interdisciplinary collaboration to address complex health challenges that transcend national and sectoral boundaries [168]. This concept recognizes that disease transmission, environmental contamination, and food safety are interconnected, emphasizing that holistic interventions across medicine, veterinary science, ecology, and environmental sciences are essential for sustainable solutions [169]. Food systems, which integrate agricultural, industrial, and environmental processes, reflect this interdependence, with foodborne illnesses and contaminants often originating from the convergence of human, animal, and environmental factors [170]. Among these threats, heavy metal (HM) pollution has become a pressing global concern due to its persistence, bioaccumulative properties, and toxicity. Industrial emissions, mining activities, pesticide use, and waste mismanagement are significant contributors to environmental HM contamination, leading to widespread deposition of toxic elements such as Pb, Cd, As, mercury (Hg), zinc (Zn), and copper (Cu) in air, water, and soil [171]. These contaminants not only disrupt ecosystem functionality but also infiltrate agricultural production systems, contaminating crops, livestock, and seafood, which then serve as key pathways for exposure in humans and animals [172]. Studies indicate that Cd and As are highly toxic even at low concentrations, and repeated exposure through consumption of contaminated food and water poses risks of chronic inflammation, organ disfunction and even carcinogenesis [171,172,173]. Such contamination is exacerbated by irrigation with polluted water, use of sewage sludge, and pesticide residues, which gradually increase soil heavy metal content, further intensifying bioaccumulation and biomagnification through the food chain [170].

The burden of HM pollution is evident in agricultural ecosystems, where deposition in soils disrupts plant physiology, microbial activity, and nutrient cycling, reducing soil fertility and crop productivity [174]. Seafood is a notable vector of human HM exposure, with As, Cd, Hg, and Pb levels often exceeding regulatory thresholds, especially in marine environments subject to industrial pollution [172]. Furthermore, meat products exhibit varying HM concentrations depending on the species and feeding environment, as demonstrated by lower levels in mutton compared to other livestock and higher Cd and Pb accumulation in the kidneys, liver, and spleen of horses [175]. These findings highlight the role of environmental conditions, industrialization, and feeding practices in shaping exposure risk. Globally, contamination of freshwater systems compounds these challenges, with an estimated 14% of the world’s population lacking access to clean drinking water, 32% lacking adequate sanitation, and nearly five million deaths annually attributable to waterborne diseases [176]. Heavy metals represent a major component of this crisis, as 23 of 35 identified toxic metals are classified as heavy metals, posing serious ecological and health threats due to their non-biodegradability and high persistence in aquatic environments [176,177]. Reports from Bangladesh, for example, demonstrate the direct health consequences of heavy metal contamination in groundwater, where elevated concentrations of As and Cd have been linked to cancer and other chronic diseases in both adults and children [173].

Animal models are essential for understanding the molecular, cellular, and ecological impacts of heavy metal exposure, making them indispensable for advancing translational research [178]. Rodent models, particularly mice and rats, are frequently employed due to their genetic and physiological similarities to humans, which make them ideal for studying toxicokinetics, toxicodynamics, and mechanistic pathways of heavy metal toxicity. Fish models, such as zebrafish and rainbow trout, serve as sensitive indicators of aquatic pollution, enabling studies of metal metabolism, detoxification, and transgenerational effects. Avian models, including Japanese quail and chickens, provide valuable insights into environmental exposure routes and the effects of metals on reproduction, development, and population-level dynamics [179]. To note, specialized studies using animal models of cadmium-induced renal toxicity have established that the proximal tubules of nephrons are the primary targets of Cd exposure, with Cd^2+^ shown to disrupt the activity of renal Na^+^/K^+^-ATPase, leading to nephrotoxicity and impaired renal function [131]. These findings underscore the need for precise model selection, which is essential for accurately extrapolating laboratory findings to humans and wildlife, thereby improving risk assessment and informing regulatory standards. The breadth of animal models available also facilitates the investigation of chronic, low-dose exposure scenarios that are often more relevant to environmental health than acute toxicity studies.

Monitoring environmental contamination is a cornerstone of One Health strategies, as early detection and intervention are crucial for mitigating the molecular effects of heavy metals on humans, animals, and ecosystems. Globally, regulatory frameworks such as the European Union’s Integrated Pollution Prevention and Control (IPPC) directive, REACH regulations, the United States Pollution Prevention Act, and China’s Environmental Protection Law emphasize pollution control, risk reduction, and corporate accountability, while over 176 countries have adopted environmental protection legislation [180]. However, enforcement remains inconsistent, particularly in low- and middle-income countries, where industrial expansion and agricultural intensification frequently outpace regulatory oversight. A One Health approach advocates for stronger collaboration between environmental, animal, and public health sectors to address these systemic challenges. Strategies include industrial emissions control, monitoring of soil, food, and water for contaminants, and adoption of sustainable agricultural practices to prevent heavy metal buildup in food systems [175]. Public education is also critical, particularly for vulnerable populations such as pregnant women and children, who are disproportionately affected by heavy metal exposure. Campaigns that promote dietary awareness, such as limiting consumption of high-mercury fish, alongside the use of personal protective equipment like masks in high-exposure areas, can substantially reduce risk [180].

Remediation of heavy metal contamination requires integrated approaches that combine multiple strategies for maximum efficacy. Methods such as adsorption with biochar, photocatalysis, electrokinetic treatments, and biological approaches like microbial and plant-based bioremediation are among the most promising solutions [181]. Phytoremediation offers eco-friendly options to stabilize, extract, or filter metals through processes like phytoextraction, phytostabilization, and rhizofiltration, reducing their bioavailability and preventing further entry into the food chain [171].

Real-world applications highlight its translational value. Field and semi-field studies using Brassica juncea (Indian mustard) have demonstrated efficient Cd and Pb uptake in contaminated agricultural soils [182], while Vetiveria zizanioides (vetiver grass) has shown strong tolerance and accumulation capacity in mixed-metal soils in regions such as Burkina Faso [183,184]. These examples illustrate how phytoremediation can simultaneously improve soil quality, reduce dietary exposure, and operationalize One Health principles in agricultural landscapes.

However, these technologies require continued research to address high costs and the risk of secondary pollution, especially in resource-limited settings (WANG). International organizations such as the United Nations and the Organization for Economic Co-operation and Development (OECD) emphasize standardized methodologies for environmental monitoring, robust data-sharing mechanisms, and the precautionary principle to guide regulatory actions even under scientific uncertainty (WANG). The convergence of scientific innovation, policymaking, and community engagement is essential to developing resilient systems capable of preventing contamination, mitigating molecular damage, and safeguarding global health in alignment with One Health principles.

## 7. Research Gaps and Future Directions

Investigating heavy metal toxicity requires well-designed experimental models, yet most studies focus on individual metals and fail to capture the combined exposures commonly encountered in real environments. Conventional approaches (i.e., in vitro, in vivo methods) provide valuable insights, but there is a growing need for models that reflect complex, multi-metal effects. In vitro systems, particularly cultured cell lines, remain essential for exploring mechanisms such as oxidative stress, DNA damage, and disrupted cellular homeostasis [185]. These systems allow precise dosing and high-throughput analysis, although research often examines metals separately. To overcome these limitations, advanced platforms such as 3D organoids, organ-on-a-chip devices, and co-culture systems better replicate tissue interactions and cumulative metal effects, improving relevance to human health. Similarly, in vivo models are crucial for evaluating systemic toxicity and organ-specific accumulation but frequently overlook interactions between multiple metals, highlighting the need for more refined experimental designs. Combining mechanistic in vitro data with in vivo toxicokinetic information provides a robust framework to understand multi-metal toxicity and supports predictive modeling, public health interventions, and One Health approaches that protect humans, animals, and ecosystems.

Another important gap concerns the limited understanding of synergistic and antagonistic interactions among Cd, As, and Pb under mixed exposure conditions. Although these metals frequently co-occur in contaminated soils, most mechanistic studies evaluate them individually, limiting the interpretation of real-world risks. Existing evidence suggests possible synergistic effects, including amplified oxidative stress, dysregulation of metal transporters, and enhanced cellular damage when metals interact. Conversely, antagonistic interactions have been observed when competition for sorption sites or uptake pathways reduces the bioavailability or toxicity of one metal in the presence of another. However, these patterns remain poorly characterized in gastric tissue models and plant systems, underscoring the need for experimental designs that incorporate realistic multi-metal exposures.

Building on these experimental insights, evidence from human populations exposed to heavy metals reveals significant biological consequences at the epigenomic level. Cd induces oxidative stress, impairs DNA repair, alters apoptosis, and acts as an endocrine disruptor; brief exposure can cause DNA hypomethylation, while chronic exposure enhances DNMT1 activity and DNA hypermethylation, reducing tumor suppressor gene expression. Cd also influences oncogene activation, miRNA expression, and signaling pathways such as Ras and NF-κB, affecting mitochondria, Wnt, and metabolic pathways [185,186,187,188,189,190,191,192,193,194]. Similarly, As alters thousands of CpG sites in blood cells, regulating genes involved in the cell cycle, morphogenesis, and neurogenesis [195,196,197,198,199,200,201,202], whereas Pb exposure similarly induces DNA methylation changes and alters microRNA expression, contributing to systemic toxicity [181,202,203,204,205,206,207].

These molecular alterations are reflected in epidemiological evidence: gastric cancer incidence increased by 8.2% in villages with As-contaminated water [208], gastrointestinal cancer rates were higher in As-polluted regions in Turkey [209], and strong correlations exist between gastric cancer and areas with Pb, As, and Sb deposits [2]. Inorganic Pb exposure is associated with gastric cancer risk (OR = 3.0, 95% CI = 1.2–7.3; OR = 2.0, 95% CI = 1.1–3.8) [210], while Cs and Pb exposure increase mortality from stomach, esophageal, and lung cancers [211]. Gastric cancer patients in Tabriz, Iran, exhibited higher urinary Cd levels (OR = 1.70, 95% CI = 1.35–2.20) [212], and elevated topsoil Pb levels were significantly linked to primary gastric cancer [213]. Collectively, these findings emphasize the critical role of epigenetic alterations in mediating the health effects of environmental metal exposure.

Despite these advances, research gaps persist. Future studies should investigate interactions between multiple metals, cumulative impacts on plant physiology, genetic variation, and heavy metal transfer in crops [29]. Moreover, sustainable remediation strategies, including bioremediation and phytoremediation, require interdisciplinary collaboration to optimize molecular- and organism-level interventions [180]. Addressing these gaps will strengthen our understanding of heavy metal toxicity and support effective strategies to protect human, animal, and environmental health.

## 8. Conclusions

Heavy metal contamination of soils, particularly with Cd, As, and Pb, represents a persistent and multifactorial threat to human and environmental health. These elements alter soil chemistry, accumulate in crops, and enter the human body through the soil–plant–food chain, where they act as molecular disruptors that promote oxidative stress, DNA damage, mitochondrial dysfunction, and epigenetic reprogramming. Within the gastric microenvironment, these metals interact synergistically with *Helicobacter pylori* infection, dysbiosis, hypoxia, and chronic inflammation, amplifying carcinogenic processes and promoting angiogenesis, epithelial–mesenchymal transition, and immune evasion.

At a broader level, metal exposure reflects systemic environmental imbalances that transcend disciplinary boundaries. The One Health framework highlights that soil pH, redox potential, and organic matter govern metal mobility and bioavailability, while agricultural practices and water management influence human exposure and disease risk. Incorporating these environmental parameters with molecular biomarkers of oxidative stress, inflammation, and genomic instability can strengthen surveillance systems and guide targeted interventions.

Advancing this field requires translational integration of environmental monitoring, molecular biology, and public health. Future research should prioritize mixed-metal exposures, cumulative risk modeling, and the use of humanized and organoid-based models to unravel early pathogenic mechanisms. In addition, translational priorities should include validating molecular biomarkers in clinical settings, developing clinical risk-stratification tools informed by environmental exposure data, and designing interventional studies that test whether reducing household or occupational metal exposure leads to measurable improvements in gastric precancerous lesions or inflammatory profiles. Strengthening these bench-to-clinic pathways will enhance the actionable impact of current evidence and support more effective prevention strategies. Coordinated soil remediation, sustainable agricultural policies, and biomonitoring of vulnerable populations are essential to reduce the burden of gastric cancer associated with heavy metal exposure and to safeguard the interconnected health of humans, animals, and ecosystems.

## Figures and Tables

**Table 1:** Main anthropogenic sources of Cd, As, and Pb and their pathways into soils.

Metal	Primary Source	Secondary Source	Entry Pathway
**Cadmium (Cd)** [45]	Phosphate fertilizers	Mining (Zn, Pb), sewage sludge	Direct application, atmospheric deposition
**Arsenic (As)** [58]	Mining (gold, copper), historic pesticides	Smelting, coal combustion	Deposition, irrigation, direct application
**Lead (Pb)** [59]	Legacy leaded fuels, mining	Metallurgy, batteries	Deposition, waste disposal

**Table 2:** Microenvironmental co-factors that interact with cadmium, arsenic, and lead to promote gastric carcinogenesis—exemplar pathways and translational readouts.

Co-Factor	Metal-Linked Effects	Principal Pathways/Processes	Representative Readouts (Research/Clinical)
***H. pylori* and dysbiosis**	Elevated ROS and RNS, epithelial barrier failure, selection for metal-tolerant and nitrosating taxa, and enhanced nitrate–nitrite–NO chemistry.	PRRs (TLRs), NF-κB/STAT3; NLRP3; nitrate–nitrite–NO axis	16S/metagenomics (nitrosating taxa); cytokines (IL-6, IL-8, TNF-α); epithelial permeability/tight-junction assays [103,104,105,106,107,108,109,110]
**Innate immunity (macrophage–epithelium crosstalk)**	Increased macrophage activation and cytokine feedback loops that favor tumor cell proliferation and survival, with accompanying epigenetic remodeling	NF-κB/STAT3; IL-6/JAK/STAT; IκBα degradation/JAK2 phosphorylation; DAMP sensing	THP-1 or primary macrophage co-cultures; phospho-STAT3, IκBα degradation; multiplex cytokine panels; ATAC-seq/ChIP for chromatin effects [111,112,113,114,115,116,117]
**Inflammasome signaling**	Metals induce mitochondrial stress, lysosomal injury, and ROS that activate NLRP3, leading to IL-1β and IL-18 maturation and driving chronic, proliferative inflammation	NLRP3–caspase-1 axis; K^+^ efflux; mtROS; MAO-B–H_2_O_2_ amplification	Pro-/mature IL-1β and IL-18; caspase-1 activity; NLRP3/ASC specks; mitochondrial dysfunction assays [103,118,119,120,121,122]
**Hypoxia and angiogenesis**	ROS inhibits PHD activity, stabilizing HIF-1α under normoxic conditions, which in turn upregulates VEGF and angiopoietins and drives glycolytic, acid-tolerant tumor phenotypes	HIF-1α stabilization; aberrant vasculature; metabolic rewiring (glycolysis/lactate)	IHC for HIF-1α/VEGF; microvessel density; lactate assays; hypoxia reporters [127,128,129,130,131,132,133,134,135]
**Stromal remodeling (CAFs/ECM/MMPs)**	Elevated MMPs; aligned collagen tracks; immune evasion; heightened oxidative tone with Cd/As; Pb alters Ca^2+^ signaling and contractility	TGF-β–HIF crosstalk; ECM remodeling; myeloid recruitment; CAF–macrophage chemokine loops	MMP zymography; second-harmonic generation (collagen); myeloid profiling; CAF/fibroblast contractility assays [129,136,137,138,139]
**Soil–crop–diet continuum; drivers: irrigation water, soil chemistry, plant uptake; additional pathways: dust inhalation, dermal contact)**	Elevated dietary Cd/As via rice and vegetables from peri-urban/mining soils; co-exposure with nitrates; occupational dust/dermal adds to dose	Speciation and bioaccessibility (pH, ionic strength, DOC); root uptake/rhizosphere chemistry; source apportionment (PMF, APCS-MLR)	Soil/crop metals; probabilistic intake (Monte Carlo); blood/tissue metal burdens; rice Cd bioaccessibility (approximately 24 percent) [154,155,156,157,158,159,160,161,162,163,164,165,166,167]

**Abbreviations**: *H. pylori*, *Helicobacter pylori*; RNS/ROS, reactive nitrogen/oxygen species; NO, nitrite oxide; PRR, pattern-recognition receptor; TLR, Toll-like receptor; NF-κB, nuclear factor kappa-B; STAT3, signal transducer and activator of transcription 3; NLRP3, NOD-like receptor family pyrin domain–containing 3; IL, interleukin; TNF-α, tumor necrosis factor alpha; JAK, Janus kinase; IκBα, inhibitor of kappa B alpha; DAMP, damage-associated molecular pattern; ATAC-seq, assay for transposase-accessible chromatin using sequencing; ChIP, chromatin immunoprecipitation with sequencing; mtROS, mitochondrial reactive oxygen species; MAO-B, monoamine oxidase B; HIF−1α, hypoxia-inducible factor−1α; ASC, apoptosis-associated speck-like protein containing a caspase recruitment domain; PHD, prolyl−4-hydroxylase; VEGF, vascular endothelial growth factor; HIF, hypoxia-inducible factor; IHC, immunohistochemistry; MMP, matrix metalloproteinase; TGF-β, transforming growth factor beta; ECM, extracellular matrix; CAF, cancer-associated fibroblast; DOC, dissolved organic carbon; PMF, positive matrix factorization; APCS-MLR, absolute principal component scores–multiple linear regression.

## Data Availability

No new data were created or analyzed in this study. Data sharing is not applicable to this article.
